# Identification and characterization of a novel glutaminase inhibitor

**DOI:** 10.1002/2211-5463.13319

**Published:** 2021-11-08

**Authors:** Henning Cederkvist, Shrikant S. Kolan, Jonas Aakre Wik, Zeynep Sener, Bjørn Steen Skålhegg

**Affiliations:** ^1^ Division of Molecular Nutrition Institute of Basic Medical Sciences University of Oslo Norway; ^2^ Department of Pathology Oslo University Hospital‐Rikshospitalet Norway

**Keywords:** GLS inhibitor, GAC, high‐throughput screening, CB839, BPTES, CD4^+^ T cells

## Abstract

In humans, there are two forms of glutaminase (GLS), designated GLS1 and GLS2. These enzymes catalyse the conversion of glutamine to glutamate. GLS1 exists as two isozymes: kidney glutaminase (KGA) and glutaminase C (GAC). Several GLS inhibitors have been identified, of which DON (6‐diazo‐5‐oxonorleucine), BPTES (bis‐2‐(5‐phenylacetamido‐1, 3, 4‐thiadiazol‐2‐yl) ethyl sulphide), 968 (5‐(3‐Bromo‐4‐(dimethylamino)phenyl)‐2,2‐dimethyl‐2,3,5,6‐tetrahydrobenzo[a]phenanthridin‐4(1H)‐one) and CB839 (Telaglenastat) are the most widely used. However, these inhibitors have variable efficacy, specificity and bioavailability in research and clinical settings, implying the need for novel and improved GLS inhibitors. Based on this need, a diverse library of 28,000 compounds from Enamine was screened for inhibition of recombinant, purified GAC. From this library, one inhibitor designated compound 19 (C19) was identified with kinetic features revealing allosteric inhibition of GAC in the µm range. Moreover, C19 inhibits anti‐CD3/CD28‐induced CD4+ T‐cell proliferation and cytokine production with similar or greater potency as compared to BPTES. Taken together, our data suggest that C19 has the potential to modulate GLS1 activity and alter metabolic activity of T cells.

## Graphical abstract

Discovery and characterization of the novel glutamine (GLS) inhibitor C19 by high‐throughput screening from Enamine. C19 inhibits activity of GLS1 and reduces proliferation and cytokine secretion from activated CD4+ T cells.

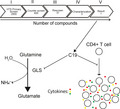

**Abbreviations**
968: 5‐(3‐Bromo‐4‐(dimethylamino)phenyl)‐2,2‐dimethyl‐2,3,5,6‐tetrahydrobenzo[a]phenanthridin‐4(1H)‐one
BPTES: bis‐2‐(5‐phenylacetamido‐1,3,4‐thiadiazol‐2yl)ethyl sulfide
C19: compound 19
DON: 6‐diazo‐5‐oxonorleucine
GAC: glutaminase C
GDH: glutamate dehydrogenase
GLS: glutaminase
HTS: high‐throughput screening
KGA: kidney‐type glutaminase
Pi: inorganic phosphate

Proliferating cells often depend on glutamine to fuel the tricarboxylic acid (TCA) cycle, generation of amino acids, nucleotides and the antioxidant glutathione [1]. The increased requirement and flux of glutamine in these cells is a condition often referred as ‘glutamine addiction’ [2, 3]. To funnel endogenous glutamine into the metabolism, cells depend on the enzyme glutaminase (GLS) (EC 3.5.1.2). In humans, two predominant isozymes of GLS exist, kidney‐type GLS encoded by the GLS1 gene, and liver‐type GLS encoded by GLS2. GLS1 and GLS2 exhibit distinct tissue distribution and are differentially regulated [4]. Endogenous GLS1 is inactive as a dimer and is activated when a tetrameric form is assembled in the presence of inorganic phosphate (Pi) [5, 6]. GLS1 converts l‐glutamine to l‐glutamate through hydrolysis of the amide group. GLS1 has two major splice variants, a long mRNA splice variant commonly referred to kidney‐type glutaminase (KGA) and a shorter form designated as glutaminase C (GAC). These two splice variants share a common N‐terminal sequence (1–550) but contain unique C‐terminal segments, 551–669 for KGA and 551–598 for GAC [7]. GAC is being the predominant isoform overexpressed in many proliferating lymphocytes, primary tumour cells and tumour cell lines as compared to normal tissues [8, 9, 10, 11, 12, 13, 14]. siRNA silencing of GAC has been shown to reduce the viability of cancer cells [15]. For lymphocytes (T and B cells), glutamine is a primary energetic nutrient and is essential for the biosynthetic processes of lymphocyte proliferation and cell cycle propagation. Moreover, lymphocytes use glutamine either similar to or greater than that of glucose [16, 17, 18]. Previously, we demonstrated that GAC is expressed in activated human T cells and glutamine deprivation and GAC inhibition attenuated T‐cell activation and clonal expansion [19, 20]. Therefore, strategies to modulate T‐cell‐mediated immune responses and cancer cell longevity are of interest for developing new treatments of autoimmune disease and improving the effectiveness of cancer therapy [20].

Several natural and synthetic compounds targeting KGA/GAC have been reported. The synthetic compound 6‐diazo‐5‐oxonorleucine (DON) is an irreversible inhibitor of the active site and binds to several glutamine dependent enzymes, and its off‐target effects are toxic to cells [21, 22]. The two GLS inhibitors BPTES and compound 968 are both allosteric inhibitors, and hence, do not compete with glutamine for the active site [23, 24, 25]. Whereas 968 binds to GLS1 in an inactive state and obstructs active tetrameric formation in the absence of Pi, BPTES seems to ‘freeze’ the enzyme in a tetrameric nonactive state independent of Pi [26, 27]. Several studies have demonstrated therapeutic potential in the low µm range of BPTES [25]. Three compounds, namely ebselen, chelerythrine and apomorphine, were identified as GLS inhibitors exhibiting 10‐ to 1500‐fold greater affinity and over 100‐fold increased inhibitory efficiency compared with DON and BPTES [28]. CB839, a GLS1 inhibitor developed by Calithera Biosciences, is the most‐studied compound in the clinical trials for the treatment of various cancers [29]. Taken together, variable efficacy, specificity and bioavailability, there is an unmet need for novel GLS inhibitors.

In the endeavour to identify novel GLS inhibitors, we screened a diverse library of 28,000 chemical compounds from Enamine by high‐throughput screening (HTS) against GAC activity. A small‐molecule GLS1 inhibitor designated as compound 19 (C19) was identified and characterized for its kinetic features *in vitro*. Furthermore, C19 was found to inhibit anti‐CD3/CD28‐induced CD4+ T‐cell proliferation and cytokine production with like or higher potency compared with BPTES.

## Material and methods

### Cloning, expression and purification of Δ129GAC

GAC DNA sequences deleted for the bases encoding amino acids 1–16, 1–72 and 1–129 were cloned and designated as Δ_16_GAC, Δ_72_GAC and Δ_129_GAC. The sequences were PCR amplified from a pET24b vector containing full length human GAC (Ensambe ID: ENST00000338435.8), 5′NdeI and 3′*Xho1* restriction sites with an N‐terminal 6x His‐tag sequence. Individual forward primers were designed as 5′3′TGCGCGCATATGCACCACCACCATCACCATAGTCCGGCAGGCGTTAG, 5′‐3′ TGCGCGCATATGCACCACCACCATCACCATAGCAGCAGCCCGAGCGAA,5′‐3′ TGCGCGCATATGCACCACCACCATCACCATAAAATCAAACAGGGTCTGCTG to generate peptides from amino acids 17‐598 and 73‐598 and 130‐598, respectively. A common reverse primer, 5′‐3′CCAGCTCGAGTTAGCTTTTTTCACCCAG was used. The pET‐ΔGAC plasmids were sequenced at Eurofins Genomics GmbH to confirm DNA sequence Δ_129_GAC construct expressed in *Escherichia coli* (*E. coli*) BL21DE3 Rosetta containing pRARE plasmid and grown in 100 mL LB medium at 37 °C with 180 rpm shaking overnight. Bacteria were inoculated in 6 L of LB medium at 30 °C until cell density was 0.6 at OD_600nm_. Δ_129_GAC overexpression was initiated by adding 0.5 mm IPTG, and after 3 h of incubation, cells were harvested. Total protein extracts were prepared by sonicating the cells in a lysis buffer containing 50 mm Tris/HCl (pH 8.5), 500 mm KCl, 40 mm imidazole and one tablet of a protease inhibitor cocktail (Roche, Indianapolis, IN, USA) followed by centrifugation for 30 min at 10,000 ***g*** at 4 °C. The first protein purification step was using a HiTrap 1 mL Ni^2+^‐NTA affinity column (Ge Healthcare Life Sciences, Chicago, IL, USA), where Δ_129_GAC was eluted using a of 0–500 mm imidazole gradient. Protein purity was analysed by SDS/PAGE in 10% gels and Coomassie brilliant blue staining (CBB). Purified protein was dialysed over night against a buffer containing a 50 mm Tris‐acetate, pH 8.6, 500 mm KCl, 10% glycerol and 5 mm TCEP (Tris(2‐carboxyethyl) phosphine hydrochloride, Sigma‐Aldrich, Milwaukee, WI, USA) and stored at 4 °C. Protein concentration was determined using the Bio‐Rad Protein Assay (Bio‐Rad, Hercules, CA, USA) with bovine serum albumin as a standard.

### High‐throughput screening enzyme assay

The assay used to determine GLS activity for HTS and enzyme kinetics measurements was adapted from Cassago *et al*. [30]. In brief, this assay determines the activity of GLS by measuring the amount of NADH produced by glutamate dehydrogenase (GDH) when converting glutamate to α‐ketoglutarate. To control for GLS‐specific activity (Δ_129_GAC activity), we tested GDH activity in the presence of 10 µm of CB839 for 2 h. This experiment demonstrates that the GLS inhibitor did not influence GDH activity [29]. For HTS, the enzyme assay was run using the following conditions: 6 nm Δ_129_GAC, 50 mm Tris‐acetate, pH 8.6, 3 units of bovine serum GDH (Sigma‐Aldrich), 2 mm NAD^+^ (Roche, Basel, Switzerland), 4 mm glutamine, 50 mm K_2_PO_4_ (MERCK, Kenilworth, NJ, USA) and 0.005% Tween‐20 (Sigma‐Aldrich). We screened 28,000 chemical compound libraries from Enamine in small‐volume 384‐well plates (Greiner Bio‐One, Monroe, NC, USA) at a compound concentration of 10 µm at the HTS Chemical Biology Screening Platform at the University of Oslo, Norway. 10 nL of each compound was applied to the wells using an ECHO 550 liquid handler (Labcyte, Sunnyvale, CA, USA) followed by dispensing and mixing of 5 µL enzyme assay mix (Hamilton Microlab Star, Reno, NV, USA). This mix was incubated at room temperature for 1 h. Columns 23 and 24 on the plate contained wells for positive and negative controls to determine *Z*‐factor values. *Z*‐factor value was calculated based on formula 1:

$$\text{Formula}\;1\;:\;z^{\prime }\;=\;\frac{3\left(\sigma p\;+\;\sigma n\right)}{\mu p\;‐\;\mu n}$$

*σp* is the SD of the positive control, *σn* is the SD of the negative control, *µp* is the mean of the positive control and µn is the mean of the negative control. Following 1‐h incubation, 5 µL of glutamine (final concentration 4 mm) was added. After 20 min, NADH was measured at ex340/em440 using a multilabel plate reader (PerkinElmer EnVision®, Waltham, MA, USA).

### Kinetic studies

Fluorescence of NADH in the HTS was measured at 340 nm after 5 min, and quantification was done by using the extinction coefficient for NADH of 6220 M^−1^·cm^−1^ and 7.7 mm of path length. The total reaction volume was 80 µL, and the measurements were performed on a FluoStar Optima (BMB Labtech, Ortenberg, Germany) microplate reader. The experiments were done in 5 replicates at 12 different substrate concentrations ranging from 0.39 to 50 mm. The kinetics were determined at compound concentrations of 16, 25 and 30 µm.

### Microscale thermophoresis analyses

For the nonlabelled microscale thermophoresis (MST) assay, a 16‐point titration series of compound was done in a protein concentration of 100 nm Δ_129_GAC. The highest concentration of titrant was 1000 µm, and the experiments were performed in 1x MST buffer (Nano Temper Technologies, München, Germany), 0.0001% DMSO and 0.1% Pluronic F‐127. Samples were loaded into Zero Background MST Premium Coated Capillaries, and measurements were done with a Monolith NT Label‐Free instrument (NanoTemper) at 25 °C using 20% MST power and 20% excitation power. For the labelled (MST), the protein (25 nm) was labelled with fluorescent dye NT‐647 according to the manufacturer’s protocol (NanoTemper) and subsequently analysed for binding with compound with a Monolith NT.115 using the same conditions as described above. For labelled analyses, MST Premium Coated Capillaries were used. The experiments were performed in duplicates and data presented as mean values ± standard deviation (SD). Data were normalized and nonlinear curve fitted using graphpad prism version 6 (La Jolla, CA, USA).

### Human CD4+ T‐cell proliferation assay

Human CD4+ T cells were isolated from buffy coats of healthy donors (obtained from Blodbanken, Oslo, Norway) using the Dynabeads CD4 Positive Isolation Kit (Life Technologies AS, Norway) according to the manufacturer's protocol. A conventional carboxyfluorescein diacetate succinimidyl ester (CFSE)‐based T‐cell proliferation was performed to test the antiproliferative effect of C19 and BPTES. In brief, CFSE (2.5 µm)‐labelled human CD4+ T cells (1 × 10^6^ cells·well^−1^) were incubated with C19 (25 µm) and BPTES (25 µm) followed by stimulation with anti‐CD3/CD28 beads (1 : 1; beads: cells ratio) (Dynabeads; Invitrogen, Waltham, MA, USA) for 96 h. After 4 days of incubation, beads were removed; cells were washed with 1x PBS, and T‐cell proliferation was assessed by measuring the CFSE dilution using flow cytometry.

### Luminex screening assay

Supernatants from T‐cell cultures were harvested after 3 days of stimulation and stored at −20 °C. Samples were diluted twofold, and the human magnetic luminex screening assay (R&D Systems, Minneapolis, MN, USA) for TNF‐α, IL‐6, IL‐10, INF‐g, IL‐4, IL‐17α, IL‐2 was performed according to manufacturer’s instructions. Data were analysed with Milliplex Analyst (Merck Millipore) and standard curves set according to manufacturer's recommendations.

### Ethics statement

Blood was donated by anonymous, healthy volunteers to the local blood bank following written informed consent in line with guidelines and approval to the Oslo University Hospital (REK‐2015/703) from the Regional Ethics Committee. It is in accordance with the standards and requirements of the Helsinki Declaration.

## Results

### Development of a robust High‐throughput Screen Assay for the identification of GLS1 inhibitors

**Fig. 1:**
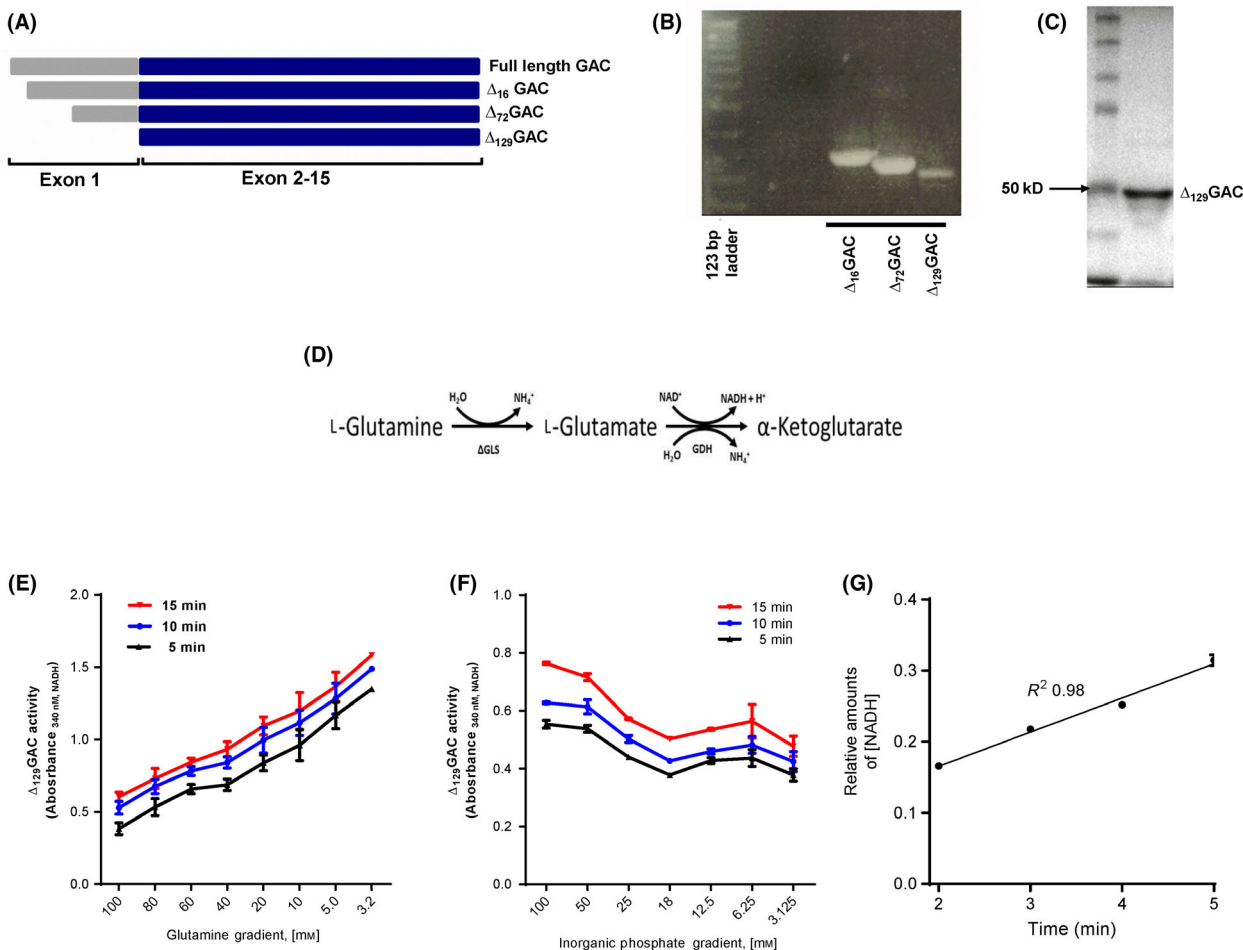
Cloning, expression, purification and functional testing of recombinant human glutaminase Δ_129_GAC. (A) Schematic representation of the cDNA of Δ_16_GAC, Δ_72_GAC and Δ_129_GAC. (B) Δ_16_GAC, Δ_72_GAC and Δ_129_GAC were PCR amplified from His‐tagged hGAC inserted in the PET24b vector. (C) Expressed recombinant His‐tagged Δ_129_GAC was purified using a HisTrap 1 mL Ni^2+^‐NTA affinity column. Protein purity analysed by SDS/PAGE shows a ~ 50 kDa His‐tag Δ_129_GAC protein. The SDS/PAGE image shown in the figure was cropped from same part of the gel. Full‐length gel image is presented in Fig. S1. (D) Δ_129_GAC activity was measured in a coupled enzyme assay where glutaminase activity was measured indirectly by monitoring Δ_129_GAC activity as the production of NADH from the conversion of glutamate to α‐ketoglutarate by GDH (see Material and Methods for details). (E) Δ_129_GAC activity was measured in a coupled enzyme assay determining NADH production as a function of glutamine conversion to glutamate by Δ_129_GAC and in turn glutamate to α‐KG with NADH by GDH. Optimal concentration of glutamine was determined by titration of incremental concentrations (3.2–100 mm) and measuring NADH at 340 nm after 5, 10 and 15 min. Glutamine concentrations above 3.5 mm led to substrate inhibition. Measured absorbance from enzyme activity was adjusted by a negative control containing only buffer, protein and substrate. (F) The optimal level of Pi in the assay was determined in the presence of 3.125–100 mm Pi. 50 mm Pi was determined as the optimal concentration for the assay. Measured absorbance from enzyme activity was adjusted by a negative control containing only buffer, protein and substrate. (G) The linearity response of the assay was determined by plotting the raw data subtracted by the blank readings to validate the assay for kinetic studies. Linearity (*R* ^2^ = 0.98) was determined in the presence of 4 mm glutamine up to 5 min, implying a robust assay. Data shown are means ± S.D (*n* = 3).

To become enzymatically active, the first 16 and 72 amino acids at the N‐terminal must be sequentially deleted in the mitochondria from endogenous GLS_GAC_ [6]. We cloned and expressed three variants of GAC, Δ_16_GAC, Δ_72_GAC and Δ_129_GAC (Fig. 1A,B). However, when expressed in bacteria, the yield of Δ_16_GAC and Δ_72_GAC protein was lower compared with Δ_129_GAC. We therefore used Δ_129_GAC in the further study. Next, Δ_129_GAC PCR product was cloned into pET‐24(b) which contained DNA encoding sequence for a His‐tag. Recombinant His‐Δ_129_GAC was expressed in *E. coli* BL21‐DE3 Rosetta bacteria to yield 2 mg protein·L^−1^ of LB medium; His‐Δ_129_GAC was purified without contamination as determined by SDS/PAGE (Fig. 1C). Using His‐Δ_129_GAC, a HTS GAC enzyme activity assay was modified according to Cassago *et al*. [30]. Briefly, the assay is a two‐step enzymatic assay where glutamine converted to glutamate by His‐Δ_129_GAC and further to α‐ketoglutarate and NADH by GDH (Fig. 1D). The glutamine consumption activity of His‐Δ_129_GAC was determined by assuming a stoichiometric ratio of one glutamine consumed to every molecule of NADH produced.

GLS1 is shown to be dependent on Pi to be activated, and the end product, glutamate, inhibits the enzyme activity [31]. Therefore, it was essential to optimize the assay for glutamine and Pi concentrations for the His‐Δ_129_GAC in the presence of GDH prior to the assay in order to acquire a suitable signal window before the screen. Incremental concentrations (3.2–100 mm) of glutamine were titrated, where NADH was measured at 340 nm after 5, 10 and 15 min at different substrate concentrations. This revealed that glutamine concentrations above 3.2 mm led to substrate inhibition (Fig. 1E). Next, we titrated Pi as the formation of the dimeric active form of human GLS is dependent on the concentration of Pi [32]. Optimal Pi concentration was titrated against 4 mm glutamine and His‐Δ_129_GAC. Again, optimal enzyme activity was measured at 5, 10 and 15 min and incremental concentrations of Pi (3.125–100 mm). The data showed highest His‐Δ_129_GAC activity at 50–100 mm Pi (Fig. 1F). Based on these experiments, we set the glutamine concentration to 4 and 50 mm Pi in the HTS assay which is also in agreement with the assay conditions mentioned by Cassago *et al*. [30]. For determining the type of inhibition in a reaction, kinetic analyses must be conducted in the linear range of the enzyme assay [33]. Linear and optimal His‐Δ_129_GAC activity was observed between 2 and 5 min (Fig. 1G). The assay showed to uphold linearity up to 5 min of continuous enzyme activity measurements (*R* ^2^ = 0.98).

### High‐throughput screening of chemical compounds inhibiting Δ_129_GAC activity

**Fig. 2:**
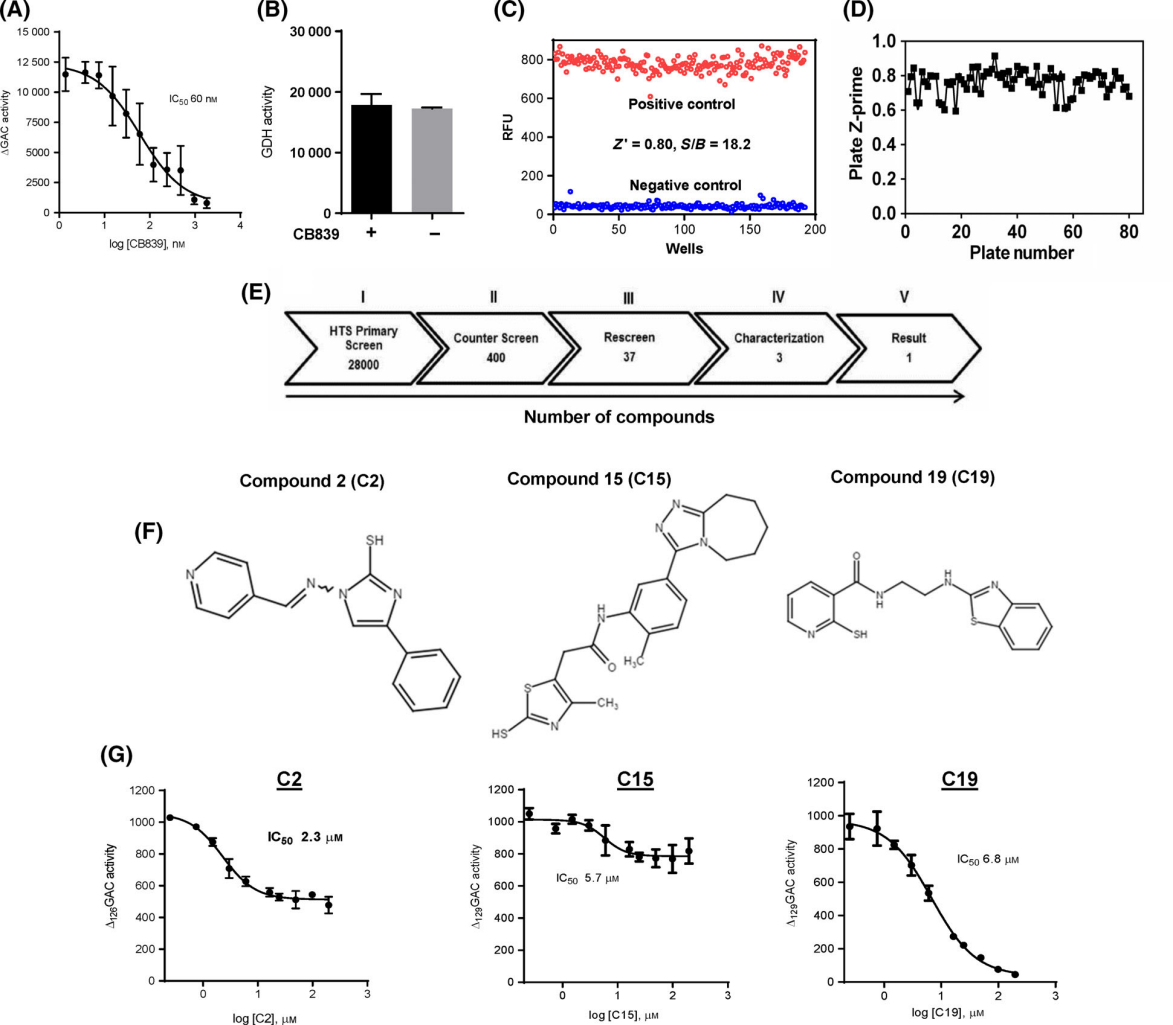
HTS of chemical compounds inhibiting Δ_129_GAC activity. (A) Incremental doses of the GLS1 inhibitor CB839 (1–1800 nm) were used as a negative control for Δ_129_GAC enzyme inhibition yielding an IC_50_‐value 60 nm by nonlinear curve fitting. Data are presented as mean ± S.D (*n* = 3). (B) GDH activity was measured after 20 min in the presence of 30 nm CB839. CB839 showed no inhibition effect on secondary enzyme (GDH) in the HTS assay. Data are presented as mean ± S.D (*n* = 3). (C) Validation of the primary screening assay. A test run of the primary screen consisting of 192 wells for both negative control (10 µm CB839, blue) and positive control (normal assay, red) was used and measured after a 20‐min time point to calculate the statistical coefficient, *Z*‐prime (*Z*′)‐factor and signal to background noise (S/B) to validate the primary HTS. (D) 28,000 compounds were measured in a total of 80 plates for Δ_129_GAC specificity. Individual *Z*′‐factor for each plate is plotted, and after 80 plate readings, it was determined to be 0.78 ± 0.07. (E) (I) through (V) show the numbers of compounds that inhibited Δ_129_GAC. This shows that 400 compounds exhibit ≥ 30% inhibitory effect in the primary screen and were submitted for counter screen (II). The rescreen (III) was performed with triplicates of 37 compounds after counter screen (step II). From the rescreen (III), 3 compounds were validated by dose response (10 points in duplicates) resulting in 3 positive compounds (IV). After characterization of 3 compounds, C19 was chosen with ≥ 90% inhibition (Step V). (F) Chemical structures of compound C2, C15 and C19, respectively. (G) Characterization of C2, C15 and C19 in the rescreen was performed by 10‐point dose response with twofold serial dilution (0.39–200 µm). IC_50_ values were determined for C2 to be 2.3 µm, C15 to be 5.7 µm and C19 to be 6.8 µm by nonlinear curve fitting. C2 and 15 in contrast to compound 19 did not inhibit Δ_129_GAC activity by 100%. Data are mean ± S.D (*n* = 3).

When performing the HTS, CB839 was used as a negative control to show full inhibition of Δ_129_GAC activity. For CB839 to be used, it requires strong binding affinity towards the primary His‐Δ_129_GAC enzyme while not interfering with the activity of secondary GDH in the coupled enzyme assay. We therefore titrated CB839 with His‐Δ_129_GAC, revealing that CB839 inhibited His‐Δ_129_GAC with an IC_50_ of 60 nm (Fig. 2A). CB839, when tested against GDH activity using glutamate in the assay, demonstrates no effect on GDH activity (Fig. 2B). Next, the HTS assay was validated by running a 384‐well plate where 50% of the wells contained positive controls (without CB839) and 50% were negative controls (with CB839). At 20 min, the assay gave a *Z*′ factor of 0.8 based upon the positive and negative controls and a signal‐to‐background ratio (S/B) of 18.2, again indicating a robust HTS assay suitable for a primary screen (Fig. 2C).

Based on these results, a diverse chemical library of 28,000 compounds from Enamine was screened where the final concentration of each compound was 10 µm. In the library screen, we obtained an average *Z*′ factor of 0.78 ± 0.07 (Fig. 2D). The primary screen resulted in the identification of 400 compounds that showed an inhibitory effect of ≥ 30% against His‐Δ_129_GAC (Fig. 2E (II)). Next, the positive compounds were counter screened against GDH activity in order to determine false positives inhibiting this enzyme instead of GLS. The counter screen resulted in 37 compounds with inhibitory effect for His‐Δ_129_GAC activity of more than 30% (Fig. 2E (III)). Positive hits were further characterized by performing a dose–response assay with a 10‐point concentration curve with duplicate data points to determine a preliminary IC_50_‐value. This step identified three compounds, designated as compound 2 (C2), compound 15 (C15) and compound (C19), respectively, that inhibited His‐Δ_129_GAC activity. The chemical structures for C2; C15 and C19 depicted in Fig. 2F. These compounds were characterized further (Fig. 2F), and it should be noted that the dose response for C19, but not C2 and C15, showed inhibition of His‐Δ_129_GAC by > 90%. In these experiments, we found that C19 inhibited His‐Δ_129_GAC in the lower micromolar range revealed as an IC_50_ of 6.8 µm (Fig. 2F).

### Mode of inhibition by C19 and determination of quantitative interaction of C19 with Δ_129_GAC

**Fig. 3:**
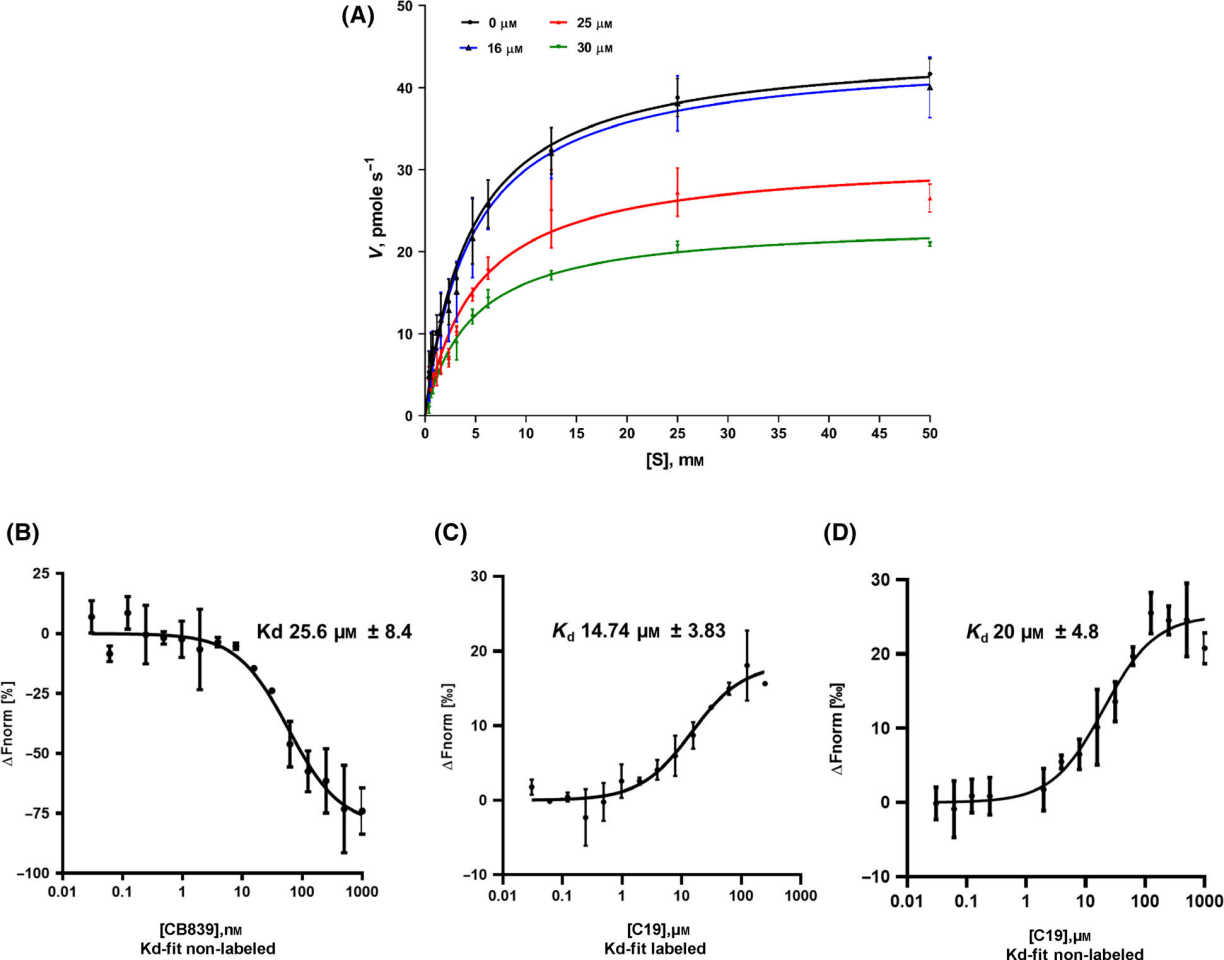
Mode of inhibition of C19 and its quantitative interaction with Δ_129_GAC. (A) Steady‐state kinetics were employed to determine the mode of inhibition of C19. Inhibitory effects were measured as enzymatic activity of Δ_129_GAC (6 nm) using the enzyme assay and in the presence of 0, 16, 25 and 30 µm of compound. *K_m_* and *V*_max_ were calculated and indicate that C19 exhibits noncompetitive inhibition as *V*_max_, but not *K_m_*, is altered by C19. The respective levels of NADH were measured after 5 min of incubation using the extinction coefficient 6220 M^−1^·cm^−1^ at 340 nm. Data points shown are mean ± SD (*n* = 5), and the curves were generated from 12 different substrate concentrations. For calculated *K_m_*, *V*_max_ and *K*_cat_ values, check Table 1. (B) Quantitative interaction between CB839 and nonlabelled Δ_129_GAC was performed by MST analysis. Incremental concentrations of CB839 (0–1000 nm) were tested with 100 nm Δ_129_GAC. Interaction of CB839 and Δ_129_GAC yielded a *K_d_*‐value of 25.6 ± 8.4 nm. (C) Using NT‐647‐labelled Δ_129_GAC (25 nm) yielded a *K_d_*‐value of 14.74 ± 3.83 µm. All *K_d_* values were determined using nonlinear curve fitting. Data are means ± SD (*n* = 3). (D) Incremental concentrations of C19 (0–1000 µm) were tested with 100 nm Δ_129_GAC yielding a *K_d_*‐value of 20.0 ± 4.8 µm. Experimental conditions were 20% MST power and 20% excitation power at 25 °C.

C19 was used for further biochemical analysis, which includes determination of mode of inhibition for recombinant His‐Δ_129_GAC. Figure 3A shows the effect of C19 on His‐Δ_129_GAC Vmax and K_m_ as a function of C19 concentration. Table 1 shows that an increasing concentration of C19 (0–30 µm) decreased *V*_max_ from 45.06 ± 0.94 to 23.63 ± 0.47 pmol·s^−1^, whereas *K_m_* was not significantly affected in these experiments. Together, this suggested that C19 acts as a noncompetitive inhibitor with respect to the substrate. Furthermore, the *k*_cat_ (enzyme substrate turnover) values for His‐Δ_129_GAC during noninhibitory events were 23.47 ± 0.4887 s^−1^ (Table 1). This value is in agreement with a previous report by Cassago *et al*. [30].

**Table 1:** Steady‐state kinetic analysis of ∆_129_ GAC in the presence of C19.

*[I]*	*V*_max_	*K_m_*	*K*_cat_
µm	pmol·s^−1^	mm	s^−1^
0	45.06 ± 0.9383	4.557 ± 0.2697	23.47 ± 0.4887
16	44.22 ± 1.1990	4.737 ± 0.3624	23.03 ± 0.6245
25	31.59 ± 1.2750	4.822 ± 0.6334	16.46 ± 0.6640
30	23.63 ± 0.4655	4.644 ± 0.2591	12.31 ± 0.2425

To further determine the biomolecular affinity of C19 for His‐Δ_129_GAC, microscale thermophoresis (MST) techniques of labelled and nonlabelled His‐Δ_129_GAC were applied. MST enables determination of the dissociation constant (K_d_) and reveals physical interaction between enzyme and compound. Due to its good solubility and selectivity, we used CB839 as a control compound in MST assay to validate the structural integrity of protein. The MST signal showed a single binding event of CB839 to His‐Δ_129_GAC with a K_d_ of 25.6 ± 8.4 nm. This is in agreement with previous results by Gross *et al*. and Stalnecker *et al*. [29, 34] (Fig. 3B). The thermophoretic movement of the C19‐His‐Δ_129_GAC enzyme–inhibitor complex was also a single binding event with a K_d_ of 14.74 µm ± 3.83–and 20.0 µm ± 4.8 for labelled – and nonlabelled MST experiment, respectively (Fig. 3C,D).

### C19 reduces anti‐CD3/CD28‐induced CD4+ T‐cell proliferation and inhibits cytokine production

**Fig. 4:**
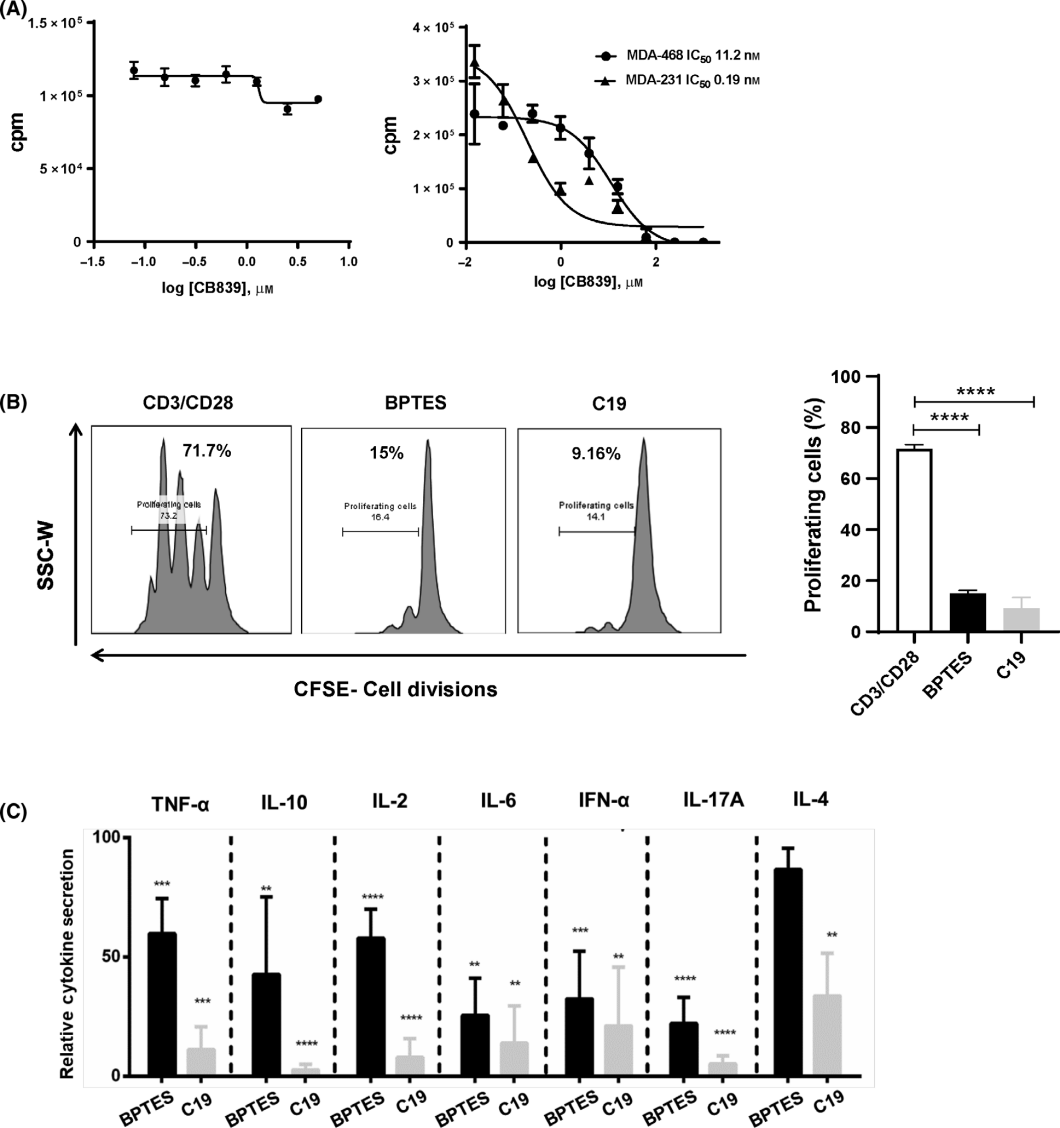
C19 inhibits anti‐CD3/CD28‐induced CD4+ T‐cell proliferation and cytokine secretion superior to BPTES. (A) Activated CD4+ T cells, MD‐468 and MD‐231 TNBC cell lines were treated with control compound CB839, and thymidine incorporation was assayed after 72 h of proliferation. Data are presented as mean ± S.D (*n* = 3). (B) CFSE‐labelled 1 × 10^6^ purified human CD4+ T cells were treated with C19 (25 µm) and BPTES (25 µm) followed by stimulation with anti‐CD3/CD28 beads for 96 h. At 96 h, cells were subjected to flow cytometry. Representative flow cytometry histograms for a single donor demonstrating proliferation of CFSE‐labelled CD4+ T cells at 96 h in culture with anti‐CD3/CD28 beads, BPTES (25 µm) and C19 (25 µm). Data are presented as the mean of 2 independent experiments ± SD run with triplicates. Measurement bars and numbers represent the percentage of proliferating cells. (C) Levels of TNF‐α, IL‐10, IL‐2, IL‐6, INF‐γ, IL‐17A and IL‐4 were measured in cell culture supernatants on day 3 of α‐CD3/CD28‐stimulated CD4+ T cells in the presence of either BPTES (25 µm) or C19 (25 µm); and data were normalized to stimulated control. All the cytokines showed significant decrease in concentration in the presence of C19 compared to control. Data are means ± SD (*n* = 9; ****P < 0.001, ***P < 0.005, **P < 0.01, *P < 0.05).

It has been shown that CB839 has minimal effect on T‐cell activation and proliferation but possess a potential in limiting breast cancer cell proliferation [29]. As shown Fig. 4A, CB839, indeed, did not inhibit CD4+ T‐cell proliferation even at doses up to 1 mm but it was able to inhibit proliferation of the TNBC, MDA‐468 and MDA‐231 with an IC50 of 11.23 and 0.19 nm, respectively [29]. We have previously shown that BPTES inhibits anti‐CD3/CD28‐induced CD4+ T‐cell proliferation in high micromolar range [19]. In order to investigate the efficacy of C19, we treated CFSE‐labelled CD4+ T cells with BPTES (25 µm) and C19 (25 µm) followed by activation with anti‐CD3/CD28 beads (ratio 1 : 1). As shown in Fig. 4B, BPTES and C19 demonstrated robust antiproliferative effect as compared to anti‐CD3/CD28‐stimulated cells (anti‐CD3/CD28‐stimulated 71.70% ± 1.60; BPTES 15.03% ± 1.21, C19‐ 9.16% ± 4.27).

Our previous study suggests that BPTES inhibits cytokine production in high micromolar range [19]. To investigate the potency of C19 in regulating cytokine production, we compared the effect of a fixed dose C19 (25 µm) to BPTES (25 µm). These data demonstrate that C19 inhibits the production of proinflammatory cytokine TNFα, IL‐6; Th1 cytokine IL‐2, INF‐γ; Th2 cytokine IL‐4, IL‐10 and Th17 cytokine IL‐17A significantly better than BPTES (Fig. 4C). In summary, GAC inhibition by C19 has higher potency for inhibiting anti‐CD3/CD28‐induced CD4+ T‐cell proliferation and cytokine production compared with BPTES.

## Discussion

In the present study, we screened a diverse library of 28,000 chemical compounds capable of inhibiting recombinant purified GAC. Compound 19 (C19) was identified, as a potential allosteric inhibitor, targeting GAC activity in the µm range in biochemical assays which possess the ability to inhibit anti‐CD3/CD28‐induced CD4+ T‐cell proliferation and cytokine production. GAC catalyses the first step of glutamine metabolism and is expressed at elevated levels in many proliferating cells including cancer cells and in immune cells involved in autoimmune diseases. Due to this, several attempts have been made to develop small‐molecule inhibitors targeting GAC activity [23, 24, 25, 29, 35, 36].

The variation in potency and specificity of the various compounds known to inhibit GLS1 may rely on a number of factors including inhibitor solubility, membrane permeability, and affinity and specificity for GLS1. Moreover, cell‐specific expression and the ratio between the various GLS1 isoforms may influence the efficacy of a given inhibitor [29]. Hence, the relative expression of GLS1 isoforms may be of particular importance since several proliferating cancer and activated immune cells (e.g. T cells) predominantly express GAC whereas nonproliferating cells express KGA [37, 38]. Initially developed glutamine analog inhibitors such as acivicin and azaserine showed antitumour effects but exhibit off‐target effects [39]. DON is cytotoxic as its off‐target effects influence the activity of several transaminases vital to amino acid metabolism. Moreover, 968, BPTES and CB839 inhibit GLS1 with varying potencies *in vitro* ranging from 2.5, 3 and 0.03 µm and with respect to cell types [23, 29, 35, 36]. Moreover, aqueous solubility and metabolic stability of BPTES and 968 are poor and these compounds have therefore proven to be unsuitable for clinical applications [37]. Based on these facts, we aimed to identify novel GLS1 inhibitors.

C19, a novel GLS1 inhibitor, was identified after a HTS using a coupled enzyme assay with an excellent *Z*′‐prime value (0.8) and a signal‐to‐background noise ratio (18.2). Of the initial 28,000 compounds in the library, 400 inhibited the coupled enzyme assay by ≥ 30% and were submitted to tests in a counter screen to determine whether they were specifically inhibiting Δ_129_GAC or instead targeting GDH. The HTS counter screen resulted in three positive hits (C2, C15 and C19), again inhibiting Δ_129_GAC ≥ 50%. C2 and C15 both were ruled out due to their poor dose dependency *in vitro* on purified recombinant GAC and because they were incapable of completely inhibiting Δ_129_GAC at the assayed concentrations. Since it remained a promising hit compound, we continued characterizing C19. Simple Michaelis–Menten kinetics performing competitive studies of Δ_129_GAC with and without C19 revealed a dose‐dependent decrease in enzymatic activity without changing the *K_m_* value, suggesting a noncompetitive inhibitory mechanism. The *K_m_* value of Δ_129_GAC without the inhibitor was 4.56 mm, which is ˜ 3 times higher than the *K_m_* of 1.6 mm for GLSGAC reported previously by Hartwick *et al*. [32]. The difference might be explained by our use of an N‐truncated enzyme (Δ_129_GAC), whereas Hartwick and coworkers used a N‐ and C‐terminally truncated GLS_GAC_ form (Δ_124_GAC Δ_551‐598_) [32]. Despite the sequence differences, Δ_129_GAC and Δ_124_GAC Δ _551‐ 598_ did show identical phosphate dependency and responded to BPTES in a similar fashion [32]. To this end, our results on Δ_129_GAC were comparable to those obtained in a previous report on Δ_124_GAC. In this case, the *V*_max_ of Δ_124_GAC and Δ_129_GAC were 45.9 and 45.1 pmol·s^−1^, respectively [30]. Cassagos substrate *K_m_s* were measured to 2.1 mm compared to 4.56 mm determined by us for Δ_129_GAC [30]. The C19 compound exhibited an IC_50_ value of 6.8 µm towards Δ_129_GAC and was shown to directly bind Δ_129_GAC both using labelled and unlabelled proteins in an MST assay and to have *K_d_* values of 14.7 and 20 µm, respectively.

Similar to tumour cells, glutamine metabolism is an absolute requirement of activated CD4+T cells, which play a key role in autoimmune disease including RA [40]. To this end, GLS1 expression has been shown to increase in fibroblast‐like synoviocytes (FLS) from RA patients and has been shown to regulate proliferation of RA‐FLS [40]. Our previous finding shows upregulation of GAC in activated proliferating CD4+ T cells [19]. We showed that C19 but not C2 and C15 inhibited CD3/CD28‐induced CD4+ T‐cell proliferation with IC_50_ of 10.98 µm. CB839 exhibits antiproliferative effect in the nanomolar range in variety of cancer cells including triple‐negative breast cancer cell lines as shown in the current study as compared to C19 (6.8 µm). However, CB839 had minimal effects on anti‐CD3/CD28‐induced T‐cell proliferation [29]. C19 on the other hand did not only inhibit CD4+ T‐cell proliferation but downregulated extracellular levels of the proinflammatory cytokines, Th1, Th2 and Th17 with higher potency compared with BPTES. The imbalance between proinflammatory and anti‐inflammatory cytokine activities favours the development and progression of many autoimmune diseases, including RA [41, 42]. In RA for instance, TNFα and interleukin‐17 (IL‐17) are principal cytokines driving inflammation and bone degradation, which are hallmarks of RA pathogenesis [43, 44]. IL‐6 promotes synovitis and joint destruction while IL‐2 is important for the proliferation of activated T cell, which are pivotal detrimental in the pathogenesis of RA as well [45, 46]. Based on this, we suggest that C19 can be developed through further molecular modifications by medicinal chemistry to modulate GLS1 activity. In this way, C19 may be developed to alter dysfunctional immune cell activity associated with autoimmunity. However, more research is required to further address the mechanisms of how C19 works on cytokine production, proliferation and *in vivo* efficacy.

Together, these data highlight C19 as a novel inhibitor directly binding to GAC outside of the active site, thus acting as an allosteric inhibitor in the µm range.

## Conflict of interest

The authors declare no conflict of interest.

## Author contributions

HC and BSS conceived and supervised the study. HC, SSK, ZS, JAW and BSS designed and performed the experiments, analysed the data and interpreted the results. HC, SSK, JAW and BSS drafted, revised and finalized the manuscript. All the authors reviewed the manuscript.

## Supporting information


**Fig. S1.** Elution profile of His‐tagged Δ_129_GAC.

## Data Availability

The authors confirm that all the data generated or analysed during this study are available within this article.
